# Salt Transport in Crosslinked Hydrogel Membranes Containing Zwitterionic Sulfobetaine Methacrylate and Hydrophobic Phenyl Acrylate

**DOI:** 10.3390/polym15061387

**Published:** 2023-03-10

**Authors:** Yi-hung Lin, Jung Min Kim, Bryan S. Beckingham

**Affiliations:** 1 Department of Chemical Engineering, Auburn University, Auburn, AL 36849, USA; 2 Department of Chemical Engineering, University of Virginia, Charlottesville, VA 22903, USA

**Keywords:** zwitterionic membranes, salt transport, phenyl acrylate, crosslinked

## Abstract

Produced water is a by-product of industrial operations, such as hydraulic fracturing for increased oil recovery, that causes environmental issues since it includes different metal ions (e.g., Li^+^, K^+^, Ni^2+^, Mg^2+^, etc.) that need to be extracted or collected before disposal. To remove these substances using either selective transport behavior or absorption-swing processes employing membrane-bound ligands, membrane separation procedures are promising unit operations. This study investigates the transport of a series of salts in crosslinked polymer membranes synthesized using a hydrophobic monomer (phenyl acrylate, PA), a zwitterionic hydrophilic monomer (sulfobetaine methacrylate, SBMA), and a crosslinker (methylenebisacrylamide, MBAA). Membranes are characterized according to their thermomechanical properties, where an increased SBMA content leads to decreased water uptake due to structural differences within the films and to more ionic interactions between the ammonium and sulfonate moieties, resulting in a decreased water volume fraction, and Young’s modulus increases with increasing MBAA or PA content. Permeabilities, solubilities, and diffusivities of membranes to LiCl, NaCl, KCl, CaCl_2_, MgCl_2_, and NiCl_2_ are determined by diffusion cell experiments, sorption-desorption experiments, and the solution-diffusion relationship, respectively. Permeability to these metal ions generally decreases with an increasing SBMA content or MBAA content due to the corresponding decreasing water volume fraction, and the permeabilities are in the order of K^+^ > Na^+^ > Li^+^ > Ni^2+^ > Ca^2+^ > Mg^2+^ presumably due to the differences in the hydration diameter.

## 1. Introduction

Wastewater containing metal ions is produced from a wide variety of commercial activities, including oil extraction, metal plating, mining, battery production, paint manufacturing, etc. [1]. The metals in these streams must be removed prior to discharge as they are hazardous to the environment and human health where they have the propensity to accumulate in the human body and induce disease [2]. Wastewater treatment is difficult; however, methods such as chemical precipitation, oxidation, ion exchange, filtration, biological treatment, and adsorption have been used to remove metal ions from aqueous solutions [3,4]. Membrane separation processes are promising unit operations for the removal of these species due to the potential for high selectivity and low energy consumption, which has led to the adoption of membrane separation technologies for the remediation of wastewater [5]. However, the long-standing issue of colloidal, organic, and other membrane fouling substantially impairs membrane functionality, limiting membrane performance and lifetime [6]. Numerous attempts have been made to modify and optimize the membrane surface to improve membrane performance, as the chemical and physical characteristics of the membrane surface play a significant role in membrane performance. Relatedly, zwitterionic polymers, which bear both positive and negative charges while being charge neutral overall, have received a great deal of attention as hydrophilic, ultra-low fouling materials that can withstand the adsorption by bacteria, cells, and protein [7]. The significant surface hydration caused by electrostatically induced hydrogen bonding in these zwitterionic polymer films is thought to be the source of the antifouling characteristics [8]. The presence of the zwitterions and thereby firmly bonded hydration layers at the surface act as physical and energetic barriers to foulant surface adhesion [9]. Zwitterions have been incorporated into polymers in various ways including as surface layers, grafted into or polymerized within the pores of filtration membranes as transport-controlling materials, and within unsupported dense polymer membranes [10,11,12,13,14,15,16,17,18,19,20]. For instance, Ni et al. investigated zwitterionic polymer films fabricated using sulfobetaine methacrylate (SBMA) or carboxybetaine methacrylate (CBMA) as the zwitterionic comonomer with poly(ethylene glycol) diacrylate (PEGDA) as the crosslinker where water uptake and permeability increased with zwitterion content, but salt permeability increased for SBMA-containing and decreased for CBMA-containing films as salt concentration increased [19]. Relatedly, Chiao et al. found the coating of porous polyvinylidene difluoride (PVDF) membranes coated with zwitterionic poly(SBMA) led to higher water flux, increased bovine serum albumin rejection, and improved antifouling performance [20].

Herein, we combine phenyl acrylate (PA), a monomer previously found to improve mechanical properties [21,22], and the commercially available zwitterionic monomer sulfobetaine methacrylate (SBMA) within crosslinked polymer films using N,N′-methylenebisacrylamide (MBAA) as the crosslinker. The physiochemical properties of a series of fabricated polymer membranes at varied PA/SBMA content—including water content, dry polymer density, and transport behavior including permeability and solubility—are characterized and discussed.

## 2. Materials and Methods

### 2.1. Material

Phenyl acrylate (PA, 97%) was purchased from Ambeed, Inc. (Arlington Heights, IL, USA). N,N′-methylenebisacrylamide (MBAA, >98%) and nickel chloride (NiCl_2_) were purchased from TCI (Tokyo, Japan). 2, 2′-Azobis(2-methylpropionitrile) (AIBN, 98%) and sulfobetaine methacrylate (SBMA) were purchased from Sigma-Aldrich Chemicals (St. Louis, MS, USA). Dimethyl sulfoxide (DMSO, ≥99.9%) was purchased from Macron Fine Chemicals (Radnor, PA, USA). Lithium chloride (LiCl), sodium chloride (NaCl), potassium chloride (KCl), magnesium chloride (MgCl_2_), and calcium chloride (CaCl_2_) were purchased from VWR (Radnor, PA, USA). Type-1 deionized water (DI water) was produced by a Waterpro BT Purification System from Labconco^®^ (18.2 mΩ·cm at 25 °C, 1.2 ppb TOC) (Kansas City, MO, USA).

### 2.2. Membrane Formation

**Figure 1:**
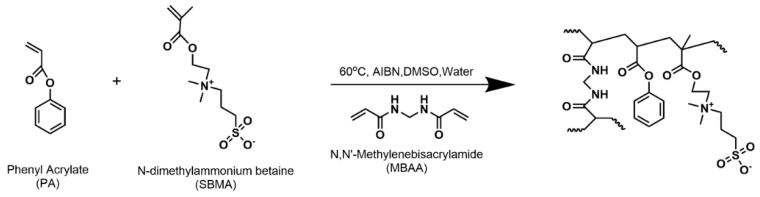
Synthetic scheme of PA−SBMA membrane.

A total of 6 membrane chemistries denoted SX-Y where X represents the mol% SBMA with the remaining PA, and Y represents the mol% MBAA of total monomer (PA and SBMA) S20-10, S20-20, S30-10, S30-20, S40-10, and S40-20 were prepared by thermal copolymerization of pre-polymerization mixtures, as shown in Figure 1.

The synthetic approach is detailed elsewhere [22]. Briefly, each pre-polymerization mixture (Table 1) contained 70 wt.% DMSO and 0.1 wt.% AIBN. It was homogenized by sonication for approximately 15 min and degassed by 10 min of nitrogen purging. These pre-polymerization mixtures were then placed between two glass plates, separated by two spacers (thickness of 0.2 mm), and incubated at 60 °C for 24 h. Finally, the as-synthesized organogels were immersed in 1 L of DI water for two days, with a daily water change to exchange DMSO for water to obtain the final hydrated membranes for characterization.

The conversion of acrylate groups in the monomers was determined through the utilization of Fourier transform infrared (FTIR) spectroscopy, using a Thermo Scientific Nicolet 6700 FT-IR spectrophotometer (Waltham, MA, USA) and OMNIC 7.3 software. The spectra wavelength range was set between 400 and 4000 cm^−1^, with a resolution of 4 cm^−1^ and 40 scans.

### 2.3. Membrane Characterization

The conversion of acrylate groups in the monomers was determined through the utilization of Fourier transform infrared (FTIR) spectroscopy, using a Thermo Scientific Nicolet 6700 FT-IR spectrophotometer and OMNIC 7.3 software. The spectra wavelength range was set between 400 to 4000 cm^−1^, with a resolution of 4 cm^−1^ and 40 scans.

### 2.4. Water Uptake, Dry Polymer Density, and Water Volume Fraction

Using a 19 mm diameter stainless-steel hole cutter, circular samples were cut from the prepared membrane films and equilibrated in DI water for at least 48 h. The samples were removed and lightly wiped to remove surface water, and their wet mass, $W_{s}$, was measured gravimetrically. Samples were then dried under a vacuum oven at 50 °C for at least 24 h prior to measuring their dry mass, $W_{d}$. Water uptake, $\omega_{w}$, was determined as follows:

(1) $$\omega_{w}=\frac{W_{s}-W_{d}}{W_{d}}\times 100\%$$

A density kit (ML-DNY-43, Mettler Toledo, Columbus, OH, USA) and scale (ML204T, Mettler Toledo) were used to measure the dry polymer density, $\rho_{p}$, as [23,24]:

(2) $$\rho_{p}=\left(\rho_{w}-\rho_{0}\right)\left(\frac{W_{d}}{W_{d}-W_{L}}\right)+\rho_{0}$$

where $W_{L}$ is the sample mass measured in the auxiliary liquid (DI water), $\rho_{w}$ is the auxiliary liquid density at the measurement temperature (997.8 kg/cm^3^ at 22 °C), and $\rho_{0}$ is the air density (1.225 kg/m^3^). By assuming the volume additivity of water and polymer [25], water uptake and density characteristics were used to calculate the water volume fraction, $\phi_{w}$, in the polymer films.

(3) $$\phi_{w}=\frac{\left(W_{s}-W_{d}\right)/\rho_{w}}{\left(W_{s}-W_{d}\right)/\rho_{w}+W_{d}/\rho_{p}}$$

### 2.5. Tensile Analysis

The mechanical characteristics of the hydrated polymer films were assessed using a commercial tensile test apparatus (DMA, TA Instruments RSA III) in the air at room temperature (~25 °C). The hydrated films (thickness of 0.2 mm) were pre-cut into rectangles measuring 10 mm by 40 mm, and each composition was characterized in at least triplicate with a 0.05 mm/s deformation rate [21].

### 2.6. Ionic Conductivity

A four-point conductivity cell (BekkTech BT-110) was used in conjunction with a Gamry Interface 1000 potentiostat to characterize the in-plane conductivity of all films at 25 °C in triplicate [26]. Films were cut into 10 mm by 5 mm rectangles and placed within the conductivity cell in DI water (500 mL), and electrochemical impedance spectroscopy (EIS) was performed after stabilizing the open-circuit potential (frequency: 10 Hz 1 MHz, AC voltage: 10 mV). Gamry Echem Analyst software was used to analyze the EIS data, and the Nyquist plot was used to determine the resistance, R (Ω). The ionic conductivity, σ, was determined as

(4) $$\sigma =\frac{L}{RWT}$$

where *L*, *W*, and *T* are the length, width, and thickness of the film, respectively.

### 2.7. Salt Permeability

A more extensive discussion of the experimental procedure can be found elsewhere [27,28]. Briefly, using temperature-jacketed (25 °C), custom-built diffusion cells outfitted with an in situ conductivity probe (PC820 Precision Benchtop, Apera Instruments, Schaumburg, IL, USA) to monitor the solute concentration in the receiver cell, the permeabilities of all films to a series of salts (NaCl, LiCl, KCl, NiCl_2_, CaCl_2_, and MgCl_2_) were measured. The receiver cell and donor cell were initially filled with DI water and a 0.1 M aqueous solution of the solute of interest (0.1 M NaCl, LiCl, KCl, NiCl_2_, CaCl_2_, and MgCl_2_), respectively. The salt permeability was calculated as follows [29,30]:

(5) $$ln\left[1-2\frac{c_{il}\left(t\right)}{c_{io}\left(0\right)}\right]=-2\frac{A}{Vl}P_{i}t$$

where $c_{il}\left(t\right)$ is the concentration of solute i in the receiver chamber at time t, $c_{io}\left(0\right)$ is the initial concentration of solute i in the donor chamber, A is the cross-sectional area (1.1423 cm^2^) for solute transport through the membrane, V is the solution volume in the donor and receiver chambers (25 mL), and l is the thickness of the membrane.

### 2.8. Solute Solubility and Diffusivity

A sorption-desorption method that has been extensively discussed elsewhere [23,28] was used to evaluate membrane solubilities for each salt of interest. Membranes were soaked in a 0.1 M aqueous solution of the salt of interest for at least three days with daily solution changes. Then the membranes were wiped dry and transferred to a known volume of desorption solution (10 mL DI water), immersed for at least three days. Finally, the concentration was determined from solution conductivity measurements and the solubility ($K_{i}$) was calculated as

(6) $$K_{i}=\frac{C_{i}^{m}}{C_{i}^{s}}$$

where $C_{i}^{s}$ is the concentration of the solute in the sorption solution and $C_{i}^{m}$ is the concentration of the solute in the film (determined from the desorption solution concentration).

The salt diffusivity, $D_{i}$ (cm^2^/s), can be calculated using the solution-diffusion model [29,30].

(7) $$P_{i}=K_{i}\times D_{i}$$

## 3. Results

### 3.1. Membrane Characterization

**Figure 2:**
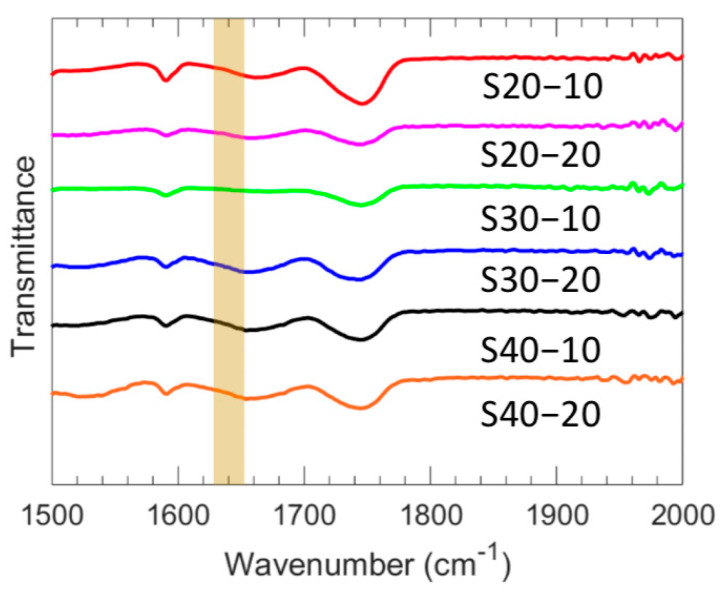
Representative FTIR spectra for each membrane composition.

**Figure 3:**
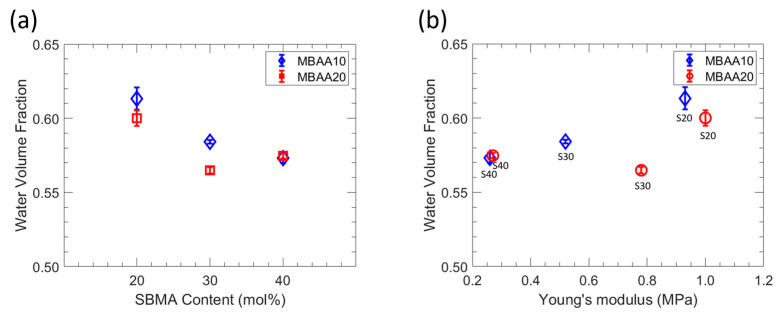
(**a**) Water volume fraction as functions of SBMA content. (**b**) Water volume fraction as function of Young’s modulus.

ATR-FTIR was utilized to analyze the zwitterionic polymer films and determine the degree of conversion of their acrylate groups. The spectra of all films are presented in Figure 2, wherein the absorption at 1640 cm^−1^ is attributed to the C=C group. Notably, this band was absent in the crosslinked film spectra, suggesting the complete conversion of acrylate groups and the breakage of the double bond during polymerization. The characterized water uptake, dry polymer density, ionic conductivity, and Young’s modulus for each of the six investigated polymer film chemistries are shown in Table 2, and the membrane water volume fraction is shown in Figure 3. With increased MBAA content, water uptake decreases for both S20-Y and S30-Y, which is attributed to the corresponding increase in crosslinking density [31,32]. For S40-Y, a decrease was observed as well, but it is within the experimental error (~1%). Water uptake also decreases with increasing SBMA content, which may be due in part to structural differences within the films. Increasing the SBMA content results in a more compact structure due to its linear shape whereas the comparably bulky phenyl ring on PA likely results in more fractional free volume, even though, structurally, PA is a more hydrophobic comonomer. In addition, we conjecture that higher SBMA content films have more ionic interactions between the ammonium and sulfonate moieties, which serve as dynamic crosslinks resulting in decreased water uptake and water volume fraction. These differences in water uptake and the polymer network structure also manifest as differences in the water volume fraction (Figure 3). For the lower MBAA content series (SX-10), the water volume fraction decreases somewhat as the SBMA content increases from 0.613 for S20-10 to 0.573 for S40-10. A similar general trend is observed for the SX-20 series. However, S30-20 showed both the lowest water uptake and water volume fraction of the membranes characterized. Relatedly, membrane ionic conductivities increase with increasing SBMA content due to the increase in ion content, which is consistent with the generally observed properties of ionic polymers [33,34]. Interestingly, the highest ionic conductivity is observed for S40-20 (1.56 mS/cm) and is significantly higher (22%) than the next highest ionic conductivity (1.28 mS/cm for S40-10). The primary difference between these two films is the increased MBAA content for S40-20; correspondingly, S40-20 has slightly lower PA and SBMA contents. The resulting structural difference is a tighter network due to the higher crosslink density for S40-20, which may be the cause of this increased conductivity. Regardless, this behavior will be investigated in a subsequent study.

### 3.2. Mechanical Characterization

**Figure 4:**
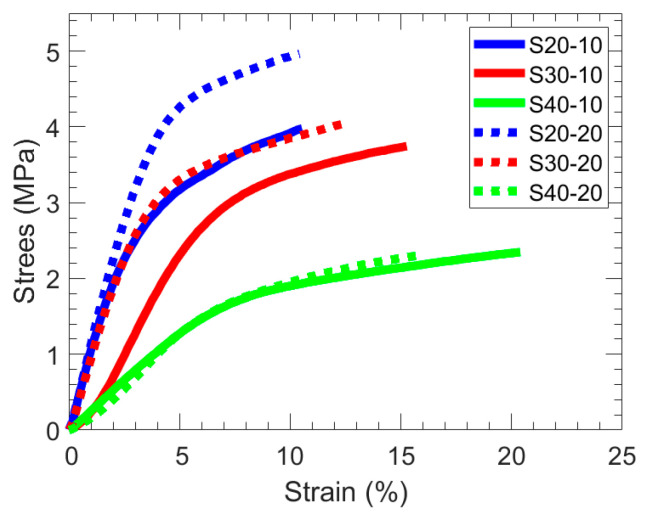
Representative stress–strain curves for (solid) SX-10 and (dotted) SX-20 films.

Tensile tests were performed in triplicate to evaluate the mechanical properties of all membranes, and exemplary stress−strain curves are shown in Figure 4. The average Young’s modulus (Table 2) increases with increasing PA and/or MBAA content. The higher Young’s modulus with increasing PA content is attributed primarily to its hydrophobicity and the rigid benzene ring, which impact the inner structure and strength of the hydrated films. However, this increase in Young’s modulus comes with a corresponding decrease in the strain at break. Notably, these SBMA-containing hydrogel membranes have a relatively high Young’s modulus compared to some other reported SBMA-containing membranes [35,36,37]. For instance, Shen et al. reported crosslinked poly(SBMA) hydrogel membranes, using MBAA as the crosslinker analogous to our work here, to have a Young’s modulus of 0.002 MPa, and crosslinked copolymer hydrogels of SBMA with acrylic acid all had Young’s moduli under 0.4 MPa [35].

### 3.3. Salt Permeability

**Figure 5:**
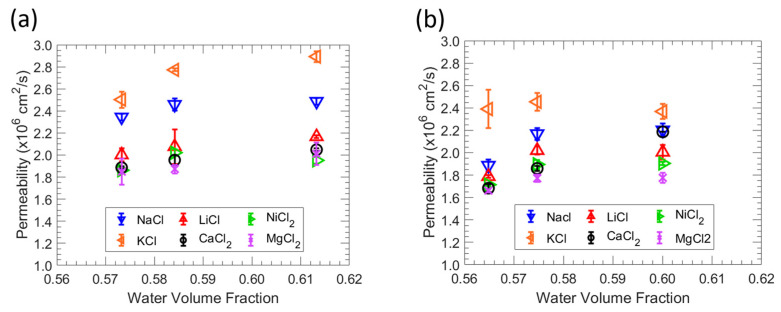
(**a**) Correlation between permeability and water volume fraction in MBAA 10 zwitterionic polymer. (**b**) Correlation between permeability and water volume fraction in MBAA 20 zwitterionic polymers. The average of three experiments is shown by each data point, and the error bars represent the standard deviation.

Permeabilities to NaCl, LiCl, KCl, NiCl_2_, CaCl_2_, and MgCl_2_ of all six films were measured by in situ conductivity diffusion cell with the results shown in Figure 5. Generally, permeability to metal ions increases with an increasing water volume fraction value or decreasing MBAA content. This behavior is expected as the water volume fraction increases as solute diffusivities tend to rise with increasing water volume fraction within a membrane (free volume theory [29,30]).

Salt permeabilities for all films generally follow the order of KCl > NaCl > LiCl > NiCl_2_ > CaCl_2_ > MgCl_2_, suggesting that the size difference between cation serves as the main criterion for differentiation since the alkali metal chloride solutions share a common anion (Cl^-^). The reported hydration radius of the metal ions [38] are in the order of K^+^ (3.31 Å) < Na^+^ (3.58 Å) < Li^+^ (3.82 Å) < Ni^2+^ (4.04 Å) < Ca^2+^ (4.12 Å) < Mg^2+^ (4.28 Å) such that we find that the observed variations in permeability between these solutions may be ascribed primarily to the identity of the cation [39]. The salt permeability of the films is consistent with water sorption behavior, where a higher water volume fraction leads to a higher permeability. The relationship between water content and salt permeability for these zwitterionic polymer membranes is generally consistent with Yasuda’s model, where high water sorption corresponds with high salt permeability.

### 3.4. Salt Solubility

**Figure 6:**
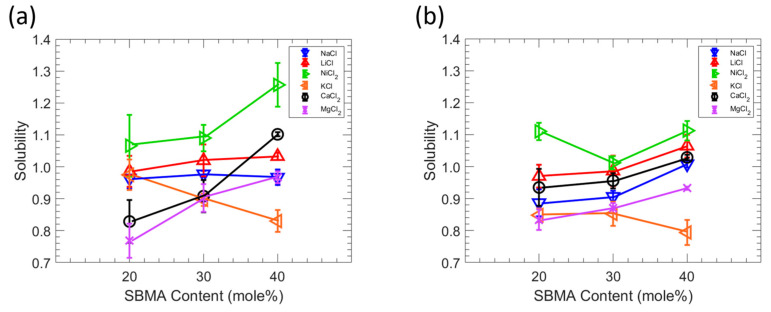
(**a**) Effect of SBMA content on the salt solubility to MBAA 10 films. (**b**) Effect of SBMA content on the salt solubility to MBAA 20 films. The average of three experiments is shown by each data point, and the error bars represent the standard deviation. Lines are guides to the eye.

The relationship between the salt sorption and the membrane zwitterion content is shown in Figure 6. Salt solubility was determined through a kinetic desorption experiment using 0.1 M solutions of the salt of interest (NaCl, LiCl, KCl, NiCl_2_, CaCl_2_, or MgCl_2_) [29,40]. Interestingly, the order of the salt sorption changes as the content of the zwitterionic monomer changes such that the salt sorption cannot be explained simply by correlating the size of the hydrated cation and surface charge density to the magnitude of salt sorption. Yasuda et al. hypothesized that the capacity of a polymer to sorb salt is severely constrained in the absence of water by assuming the water in the membrane phase dissolves all salt [29]. Since water molecules cluster at or near polymer chains in membranes with greater water absorption, direct interactions between polymer chains and ions are restricted [41]. However, as the membrane water content declines, increasing the interactions between polymer and ions, as well as the interactions between the polymer and water, may cause the ions in the membrane to behave in a highly non-ideal manner, which would then influence ion sorption. Because the water content decreases with increasing SBMA content, interactions between the polymer and the ion and the polymer and the water are thought to have an impact on how different salts interact with them within the film. In other words, the main determinants of alkali metal chloride sorption in polymers are the polymer water content and the interactions between ions and polymer segments. In addition, ion charge density, which affects an ion’s propensity for hydration and interactions with the polymer, has been shown in several investigations to affect the precise interactions between various ions and polymer segments [42,43]. The sorption of alkali chlorides is significantly impacted by the cations since NaCl, LiCl, KCl, NiCl_2_, CaCl_2_, and MgCl_2_ all share Cl^-^, a large, low-charge density ion that weakly binds water molecules. Moreover, ion charge density increases as the ion crystal radius decrease in the alkali metal ion series, where the order of the crystal’s radius is K (1.33 Å) > Ca (0.99 Å) > Na (0.95 Å) > Ni (0.70 Å) > Mg (0.65 Å) > Li (0.60 Å) [38]. The amount of water absorbed by the polymer varies depending on the salt solution because ions with various charge densities carry different amounts of water molecules into the polymer matrix. For example, Li^+^ ions carry more water molecules than K^+^ ions into the polymer matrix. Since salt sorption in charged polymers is strongly correlated with properties related to the charged groups on the polymer [44,45,46], increasing the zwitterionic monomer content generally increases the sorption such that charged groups in these zwitterionic polymer membranes may enhance salt sorption, though in different degrees as observed here [8,47].

### 3.5. Salt Diffusivity

**Figure 7:**
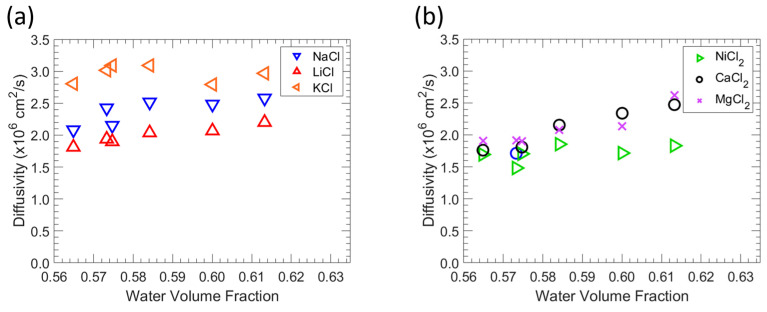
Correlation of diffusivity and water volume fraction in zwitterionic polymers. (**a**) Effect of water volume fraction on the monovalent salt diffusivity. (**b**) Effect of water volume fraction on the divalent salt diffusivity.

Salt sorption and permeability parameters were assessed and utilized to determine the effective diffusivity using Equation (8). Figure 7 presents the correlation between diffusivity and the water volume fraction. The diffusivity, $D_{i}$, of solute i in a membrane is closely related to the fractional free volume of membranes by

(8) $$D_{i}=\alpha_{i}\;\exp\left(\frac{-b_{i}}{v_{f}}\right)$$

where $\alpha_{i}$ and $b_{i}$ are empirical parameters; $\alpha_{i}$ is related to a geometric factor and the velocity of solute i, and $b_{i}$ is linked to the Lennard–Jones diameter of solute i [25,48]. 

According to Equation (8), the diffusivity of a solute of a specific size rises with $v_{f}$. Because the water volume fraction is directly connected to the fractional free volume available in the hydrated polymers for diffusion, raising the water volume fraction values generally results in higher salt diffusivity [49,50]. For a wide range of polymers, this result is consistent with a connection between salt diffusivity and water absorption. The order varies with polymer water content, indicating that ion hydration and particular interactions between the polymer and ions affect the alkali metal chloride diffusion behavior in polymers. Ions with lower hydrated radii diffuse in aqueous solutions more quickly than ions with higher hydrated radii [51].

## 4. Conclusions

This study investigates the fundamental transport properties of small molecules including NaCl, LiCl, KCl, NiCl_2_, CaCl_2_, and MgCl_2_ in a series of zwitterionic polymers prepared via thermal free-radical polymerization from SBMA and PA, which were crosslinked at two different MBAA contents. The properties of zwitterionic polymer films were investigated through various analytical techniques, including ATR-FTIR spectroscopy, water volume fraction measurements, ionic conductivity, Young’s modulus, and salt permeability. The results show that the degree of conversion of acrylate groups was high in crosslinked films and increasing the SBMA content led to a decreased water volume fraction and increased ionic conductivity due to corresponding increases in polymer density, dynamic crosslinks, and increased ion content. The higher Young’s modulus with increasing PA content was attributed to its hydrophobicity and rigid benzene ring. Interestingly, while permeabilities are generally linked to the water content within the membrane, the order of the preference for salt sorption changes as the content of the zwitterionic monomer changes. Salt permeability was primarily affected by the size of the cation, and the relationship between water content and salt permeability was consistent with Yasuda’s model. The salt sorption capacity of the polymer films could not be solely explained by the size of the hydrated cation and surface charge density but is indicative of more complex interactions between the membrane and the solutes, which requires further investigation to both understand and exploit the design of polymer membranes for emerging applications.

## Figures and Tables

**Table 1:** Pre-polymerization mixture compositions.

Membrane	PA (mol%)	SBMA (mol%)	MBAA ^1^ (mol%)
S20-10	80	20	10
S20-20	80	20	20
S30-10	70	30	10
S30-20	70	30	20
S40-10	60	40	10
S40-20	60	40	20

^1^ MBAA = mol of MBAA/(mol of PA + mol of SBMA) × 100%

**Table 2:** Water uptake, dry polymer density, and ionic conductivity of all films.

	$\omega_{w}$ (g Water/g Dry Polymer)	$\rho_{p}$ (g/mL)	Ionic Conductivity (mS/cm)	Young’s Modulus (MPa)
S20-10	125 ± 2	1.26 ± 0.04	1.20 ± 0.01	0.93 ± 0.03
S20-20	116 ± 4	1.30 ± 0.04	1.22 ± 0.04	1.00 ± 0.00
S30-10	108 ± 0	1.29 ± 0.00	1.26 ± 0.03	0.52 ± 0.02
S30-20	100 ± 1	1.29 ± 0.01	1.27 ± 0.02	0.78 ± 0.05
S40-10	104 ± 1	1.29 ± 0.01	1.28 ± 0.03	0.26 ± 0.01
S40-20	103 ± 1	1.31 ± 0.01	1.56 ± 0.03	0.27 ± 0.02

## Data Availability

Data not contained within this manuscript are available upon direct reasonable request to the corresponding author.
